# Supplementary material for: Chromothripsis during telomere crisis is independent of NHEJ, and consistent with a replicative origin
Source: Genome Res. 2019 May;29(5):737–49. doi: 10.1101/gr.240705.118 (PMC6499312; doi:10.1101/gr.240705.118)
Supplement: Supplemental Material [file supp_gr.240705.118_Supplemental_file_1.zip › contigs/annotated_contigs/DB57/contig.4.DB57_length_399_mean_cov_5.63909774436.docx]

**DB57_length_399_mean_cov_5.63909774436**

```
TCATTTGTGTCCAGACCTGCAGATTTACCTTCACCTAATTAAAGATACATTAATTAAATTAGAT|C|TCTCTGGAGAACCCTGACTAAT
 >chr7:147333075-147333139 - E=5e-27                              >chr17:67582775-67582
ACACT|GAAATGATGGCTTCTACGGCTGGAAATGTGCAAGATGAAAGATGAGCCAGGAACATATC|TTAAATAT|TCTGTTACTTTTCT
802 + E=1e-04  >chr7:147335307-147335366 - E=5e-24                       >chr7:1441672
ATTTTCTTATTATCCTCTTAACTATTTTCCAGGCTGAGTAAATTCTTTGTACACTGGATATGTGGCTAAATCAAAACAGAAAGTAGATC
49-144167489 - E=5e-130
CTTCTATCTTTGCATTTTAGAAATGAGTAACACATATCACATACCAGAGTGTATCTTTTGCAATTATTCACAAGCAACATGTTTGAAGG

TTGAACCACATACAGTCAATTGTACTGTTAAAAATTTAATGCTTTTGC
```
